# Co-Administration of IL-1+IL-6+TNF-α with *Mycobacterium tuberculosis* Infected Macrophages Vaccine Induces Better Protective T Cell Memory than BCG

**DOI:** 10.1371/journal.pone.0016097

**Published:** 2011-01-19

**Authors:** Vijender Singh, Shweta Jain, Uthaman Gowthaman, Pankaj Parihar, Pushpa Gupta, Umesh D. Gupta, Javed N. Agrewala

**Affiliations:** 1 Immunology Laboratory, Institute of Microbial Technology, Chandigarh, India; 2 National JALMA Institute for Leprosy and Other Mycobacterial Diseases, Agra, India; Universita di Sassari, Italy

## Abstract

BCG has been administered globally for more than 75 years, yet tuberculosis (TB) continues to kill more than 2 million people annually. Further, BCG protects childhood TB but is quite inefficient in adults. This indicates that BCG fails to induce long-term protection. Hence there is a need to explore alternative vaccination strategies that can stimulate enduring T cell memory response. Dendritic cell based vaccination has attained extensive popularity following their success in various malignancies. In our previous study, we have established a novel and unique vaccination strategy against *Mycobacterium tuberculosis* (*M. tb*) and *Salmonella typhimurium* by utilizing infected macrophages (IM). In short-term experiments (30 days), substantial degree of protection was observed. However, remarkable difference was not observed in long-term studies (240 days) due to failure of the vaccine to generate long-lasting memory T cells. Hence, in the present study we employed T cell memory augmenting cytokines IL-1+IL-6+TNF-α and IL-7+IL-15 for the induction of the enhancement of long-term protection by the vaccine. We co-administered the *M. tb* infected macrophages vaccine with IL-1+IL-6+TNF-α (IM-1.6.α) and IL-7+IL-15 (IM-7.15). The mice were then rested for a reasonably large period (240 days) to study the *bona fide* T cell memory response before exposing them to aerosolized *M. tb*. IM-1.6.α but not IM-7.15 significantly improved memory T cell response against *M. tb*, as evidenced by recall responses of memory T cells, expansion of both central as well as effector memory CD4 and CD8 T cell pools, elicitation of mainly Th1 memory response, reduction in the mycobacterial load and alleviated lung pathology. Importantly, the protection induced by IM-1.6.α was significantly better than BCG. Thus, this study demonstrates that not only antigen-pulsed DCs can be successfully employed as vaccines against cancer and infectious diseases but also macrophages infected with *M. tb* can be utilized with great efficacy especially in protection against TB.

## Introduction

In 1992, nearly 100 million children received BCG [1]. Despite the reality that more people have been immunized with BCG than any other vaccine, TB continues to kill some 2 million people annually and 2 billion people worldwide are infected with *M. tb* [2]. Hence, the protective efficacy of the BCG vaccine remains doubtful. The wide spread of TB has been further aggravated by the emergence of multidrug-resistant (MDR) strains of mycobacteria and the AIDS-pandemic [3]. Therefore, there is a serious need and challenge for scientific community to develop alternative vaccines for the control of the disease.

Approximately one-third of the world population is infected with *M. tb*, but only 5%–10% contract active disease while remaining develop effective immunity [2]. Further, mice cleared of TB infection respond vigorously upon re-exposure to mycobacteria and remain protected [4]. This suggests that infection with viable mycobacteria develop effective and long-lasting protective immunity. There may exist a possibility that growing bacilli express discrete molecules inside the macrophage [5], that may act as protective antigens necessary for the generation of effector and memory T cells responsible for protective immunity. Whether such protective antigens of *M. tb* that can be exploited as vaccines, still remains to be identified. [4], [5]. Incidentally, the antigens isolated from *in vitro* cultures have failed to generate considerable protective immunity, corroborating to the above mentioned hypothesis [6], [7].

In the past few decades, a number of new vaccine approaches like naked DNA vaccines, live attenuated vaccines and subunit vaccines have been elucidated. Unfortunately, none of them worked successfully against TB since they failed to generate long-lasting memory cells [8]. This indicates that novel and unique vaccination strategies still need to be explored for TB.

Recently, many studies have highlighted the role of cell-based vaccines *viz.* antigen-loaded DCs to evoke protective T cell responses against cancer, infectious diseases, etc. [9], [10], [11]. Like-wise, this also encouraged us to generate a vaccine by culturing live *M. tb* in host macrophages. Our assumption that this approach may be effective, stems from the fact that the bacterium in its natural habitat (macrophages) is likely to secrete unique antigens that may eventually help in generating protective immune response [5]. The preparation was made safe for immunization, by drug treatment [5]. Noteworthy observations obtained on vaccination with the infected macrophages was that it augmented T cell proliferation, IFN-γ production and reduction in mycobacterial load, the parameters that are crucial for protection against *M. tb*. This vaccination strategy worked successfully not only for TB but also against *S. typhimurium* [5]. This study demonstrated significant protection against both the intracellular pathogens in a short-term study (30 days). However, no CD8 T cell responses were examined and efficacy of vaccine was also not compared with BCG. The vaccine failed to generate protective immunity in long-term study (240 days).

Immunological memory can be enhanced by the use of selected cytokines [12], [13]. Hence, supplementing cytokines with vaccines to bolster T cell memory can be an attractive approach. Role of inflammatory and common γ-chain cytokines has been highlighted in the literature in enhancing T cell memory response. Cytokines like IL-1, IL-6 and TNF-α help in the expansion and survival of memory T cells [14], [15] whereas common γ-chain cytokines, IL-7 and IL-15 have dominant roles in generation and homeostasis of memory T cells [12], [16], [17].

The present study was conducted to check whether vaccination with *M. tb* infected macrophages supplemented with T cell memory enhancing cytokines could generate enduring T cell memory. We vaccinated mice with *M. tb* infected macrophages vaccine with IL-1+IL-6+TNF-α (IM-1.6.α) and IL-7+IL-15 (IM-7.15) to study the generation and sustenance of long-term (240 days) protective immunity against *M. tb* [18]. Interestingly, IM-1.6. α demonstrated considerable augmentation in both CD4 and CD8 T cell memory pool, as evidenced by significant improvement in immune response and protection against *M. tb.* Importantly, the vaccine showed better efficacy than BCG and hence can be a potent future vaccination strategy against TB.

## Results

### Vaccination with sIM-1.6.α elicits enduring Th1 response

**Figure 1:**
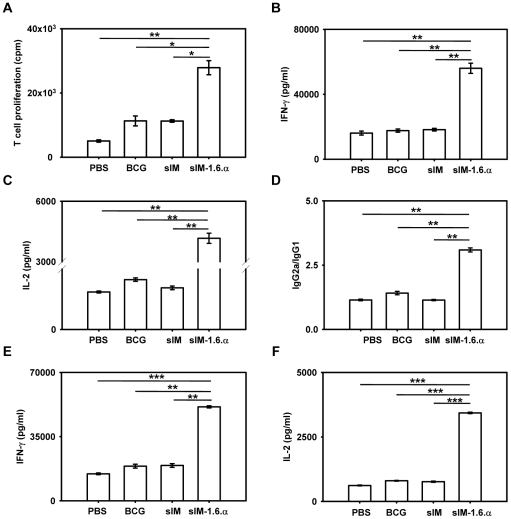
sIM-1.6.α elicits a long-lasting Th1 immune response. Mice were vaccinated with sIM-1.6.α and sIM. After 240 days, they were aerosol challenged with *M. tb* and were sacrificed 35 days later. Lymphocytes Pooled from spleen and lymphnodes of immunized mice were stimulated *in vitro* with PPD (50 µg/ml) for 48 h. T cell proliferation was monitored by ^3^H-thymidine incorporation (A); secretion of IFN-γ (B) and IL-2 (C) in the culture SNs by ELISA. Ratio of PPD specific IgG2a/IgG1 antibodies were estimated in serum (1000× dilution) (D); production of IFN-γ (E) and IL-2 (F) was estimated in the culture SNs of lymphocytes isolated from lungs, *in vitro* stimulated with PPD (50 µg/ml). Data shown are mean ± SEM and representative of two experiments with n = 3 animals per group. ‘*’, ‘**’ and ‘***’ indicate p<0.05, p<0.01 and p<0.001 respectively.

Induction of long-lasting immunity is a cardinal feature of a successful vaccine. Hence in the present study, we investigated how enduring T cell memory can be elicited by *M. tb* infected syngeneic macrophages (sIM). It is reported that combination of either IL-1+IL-6+TNF-α or IL-7+IL-15 can induce long-term T cell memory response [14], [16], [19]. Therefore, we co-administered these cytokines with the vaccine and studied the sustenance of long-lasting memory T cells. After vaccination, the mice were rested for sufficiently long-duration (240 days) before challenge with *M. tb*, to establish *bona fide* T cell memory response [18]. Interestingly, co-administration of IL-1+IL-6+TNF-α with *M. tb* infected syngeneic macrophages (sIM-1.6.α) considerably improved the immune response (Figure 1). The T cell response observed with sIM-1.6.α immunization in response to PPD (purified protein derivative from *M. tb*), was significantly better than BCG (p = 0.0253) and sIM alone (p = 0.0176) (Figure 1A). Cells obtained from the control groups inoculated with PBS (11020±643 cpm), uninfected syngeneic macrophages (6513±417 cpm), and 1.6.α alone (6981±293 cpm) failed to improve T cell response. Further, these results were substantiated using two other *M. tb* antigens, short-term culture filtrate (STCF-H37Rv) and infected macrophages cytosolic (IMC) antigenic proteins (Figure S1A and Figure S2A). However, we did not observe any change when IL-7 and IL-15 were administered along with sIM (data not shown). Hence, subsequent experiments were conducted with vaccine supplemented with IL-1+IL-6+TNF-α.

Th1 cells are known to play crucial role in protection against TB [20]. Hence we next monitored the Th1 cytokines (IL-2 and IFN-γ) elicited by the vaccine. As compared to BCG and sIM, vaccination with sIM-1.6.α showed predominance of Th1 immune response as evident by significantly higher production of IFN-γ (p = 0.0071; p = 0.0071 respectively) and IL-2 (p = 0.0057; p = 0.0052 respectively) on *in vitro* stimulation with either PPD (Figure 1B, C) or STCF-H37Rv (Figure S1B, C) or IMC (Figure S2B, C). It is a well established fact that B cells produce mainly IgG2a and IgG1 on interaction with Th1 and Th2 cells respectively [21]. Interestingly, we detected primarily *M. tb* specific IgG2a-isotypes (BCG vs sIM-1.6.α, p = 0.0037) (Figure 1D). This further substantiates the predominance of Th1 response elicited by sIM-1.6.α. Adaptive immune response is initiated in the local lymph nodes, effectors subsequently migrate to the site of infection [22], [23], [24]. Since the appearance of adaptive immunity in the lungs play a seminal role in imparting protection against *M. tb*, it was imperative to monitor the local immunity as well. Like lymphoid organs, mainly Th1 response was also observed in the lymphocytes isolated from the lungs (Figure 1E, 1F; Figure S1D, S1E; Figure S2D, S2E). No change was observed in the secretion of IL-4 and IL-5 (Figure S3). The proliferation and cytokines data signify that vaccination with sIM-1.6.α significantly evokes long-lasting Th1 immunity against *M. tb*.

### sIM-1.6.α induces enduring CD4 and CD8 T cell memory response

**Figure 2 pone-0016097-g002:**
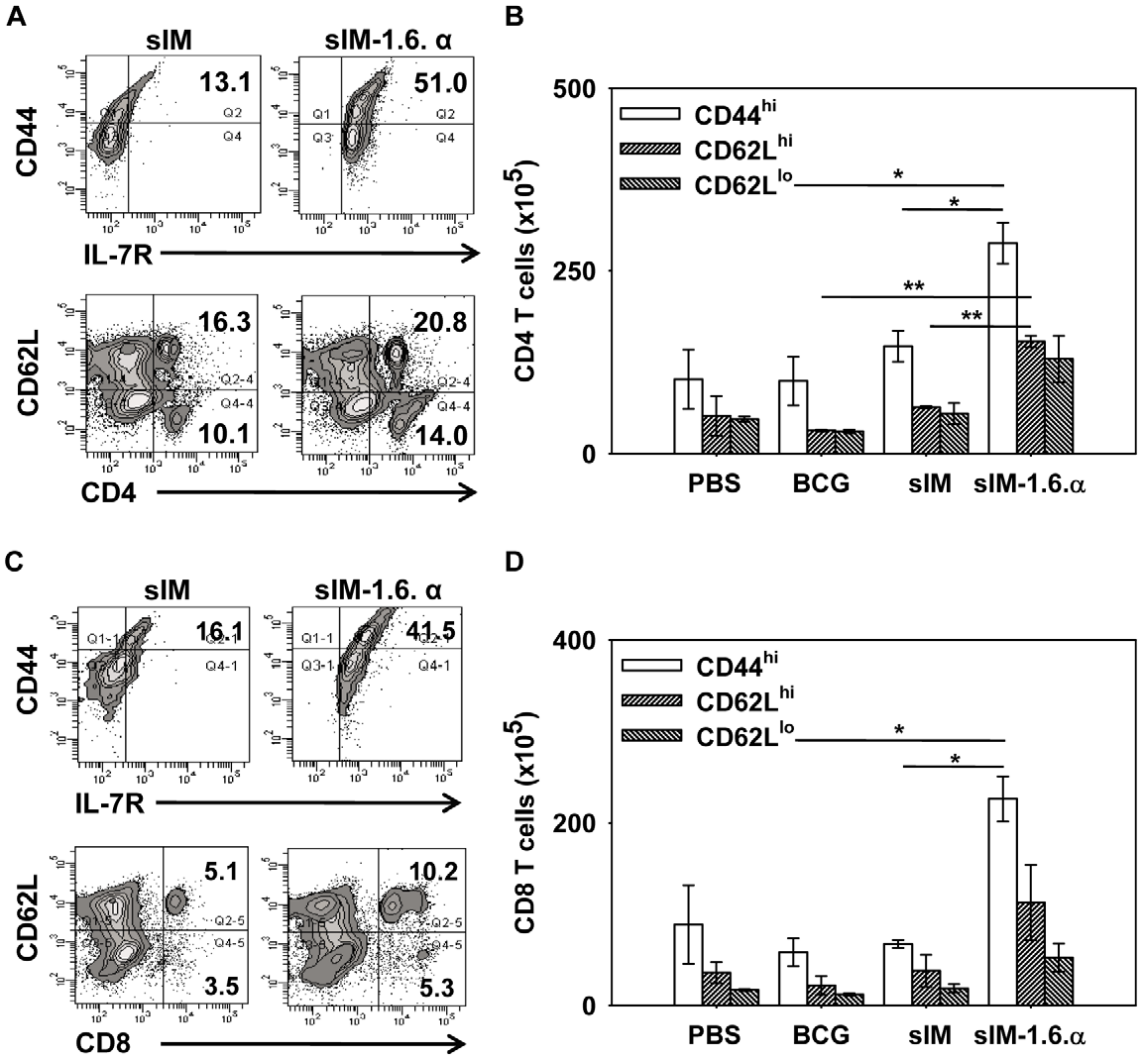
sIM-1.6.α induces enduring CD4 and CD8 T cells memory response. Mice were immunized and aerosol challenged as mentioned in Figure 1. Lymphocytes were stimulated *in vitro* with PPD (50 µg/ml) for 48 h and later stained with fluorochrome conjugated antibodies and analyzed by flowcytometry. A, flow contours depict percentage of gated CD4 T cells expressing IL-7R and CD44 (upper panel); CD62L on CD4 T cells (lower panel). C, gated CD8 T cells expressing IL-7R and CD44 (upper panel) and CD62L on CD8 T cells (lower panel). Bar diagrams show total number of CD4 (B) and CD8 (D) T cells expressing CD44^hi^, CD62L^hi^ and CD62L^lo^. Data shown are mean ± SEM of two independent experiments with n = 3 animals per group. ‘*’ and ‘**’ indicate p<0.05 and p<0.01 respectively.

We next monitored the expression of memory T cell markers viz. CD44, CD62L and IL-7R (CD127), on the cells isolated from spleen and lymph nodes of the immunized mice (Figure 2). The cells were *in vitro* stimulated with PPD. sIM-1.6.α substantially expanded pool of CD44 and IL-7R expressing CD4 T cells (13.1 *vs* 51.0%) compared to sIM vaccine (Figure 2A). Similar results were exhibited by CD8 T cells (16.1 *vs* 41.5%) (Figure 2C). Further, this observation was also supported by improvement in the central (CD44^hi^ CD62L^hi^) as well as effector (CD44^hi^ CD62L^lo^) memory T cells (Figure 2A–D). Enhancement in total number of cells was also observed (Figure 2B, D). Overall these findings specify that immunization with sIM-1.6.α can induce long-lasting memory response in both CD4 and CD8 T cells.

### Vaccination with sIM-1.6.α imparts better protection than BCG

**Figure 3 pone-0016097-g003:**
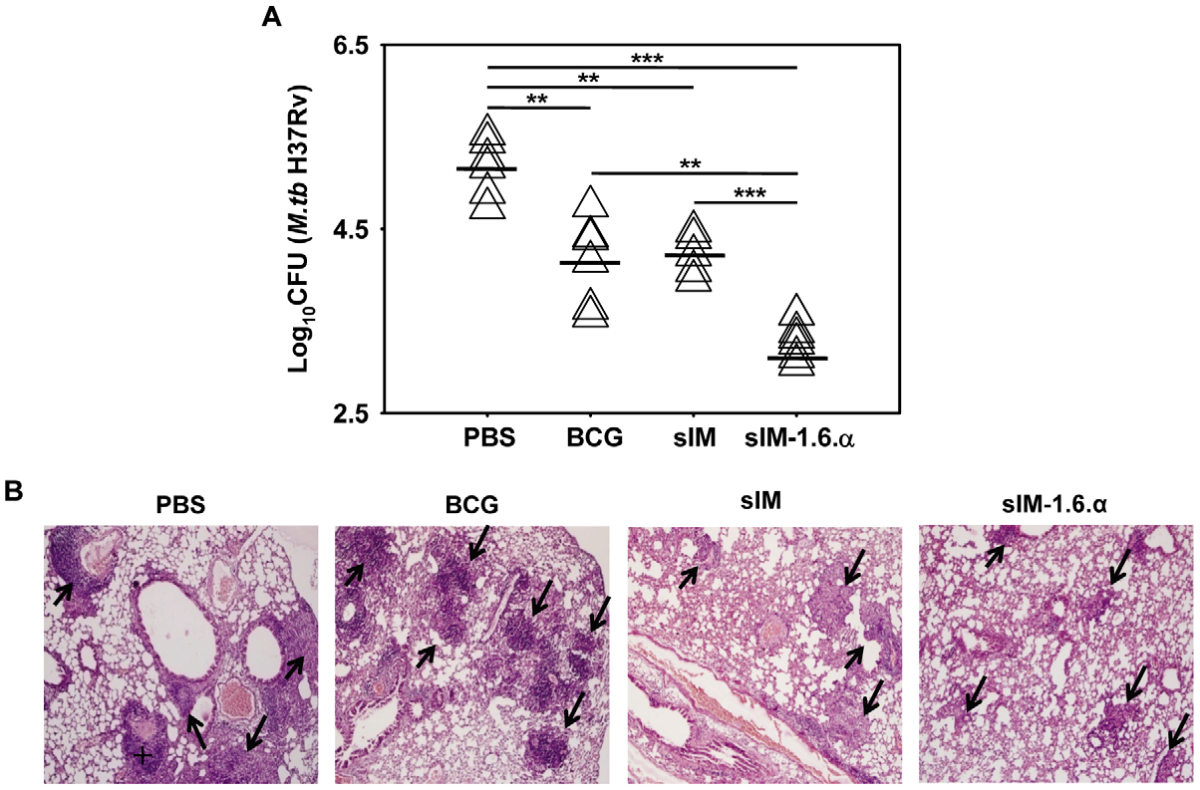
sIM-1.6.α provides significantly better protection than BCG. Mice were immunized and aerosol challenged as mentioned in Figure 1. Mycobacterial load in the lungs was enumerated by CFU plating of diluted lung homogenates. A, data are represented as log_10_ CFU (n = 6 animals/group) where each triangle indicates single mouse. B, photomicrographs (10×) of formalin fixed lungs sections stained with H & E. **‘**+**’** indicates active TB lesion with edematous follicular reaction while ‘→’ shows small or large developing follicular granulomas. Data are representative of two independent experiments. ‘**’ and ‘***’indicate p<0.01 and p<0.001 respectively.

sIM-1.6.α elicited enduring CD4 Th1 and CD8 T cell memory, the parameters favorable for a successful TB vaccine. Hence, we next checked the protective efficacy of sIM.1.6.α in *M. tb* aerosol challenged mice. Interestingly, vaccination with sIM-1.6.α considerably reduced the lung mycobacterial burden compared to sIM (p = 0.0004) and BCG (p = 0.0067) (Figure 3A). Control groups inoculated with placebo; PBS (log_10_ CFU 5.17±0.16) (Figure 3A), uninfected syngeneic macrophages alone (log_10_ CFU 5.37±0.029), and 1.6.α alone (log_10_ CFU 4.66±0.064) (Figure S4) failed to show any significant decline in CFU (p>0.05 for PBS *vs* 1.6.α or uninfected syngeneic macrophages). Interestingly, neither sIM-7.15 nor BCG-1.6.α could provide better protection than controls syngeneic uninfected macrophages (sMac) or BCG respectively (Figure S4). Further, protection imparted by sIM-1.6.α vaccination was reflected by qualitative analysis of lungs pathology. Alleviation in pathology with normal alveolar structure was observed with minimum lungs tissue involvement. In contrast, mice inoculated with BCG and placebo (PBS) revealed granulomatous infiltrations with active TB lesions (Figure 3B).

### Pragmatic approach to utilize IM-1.6.α

**Figure 4 pone-0016097-g004:**
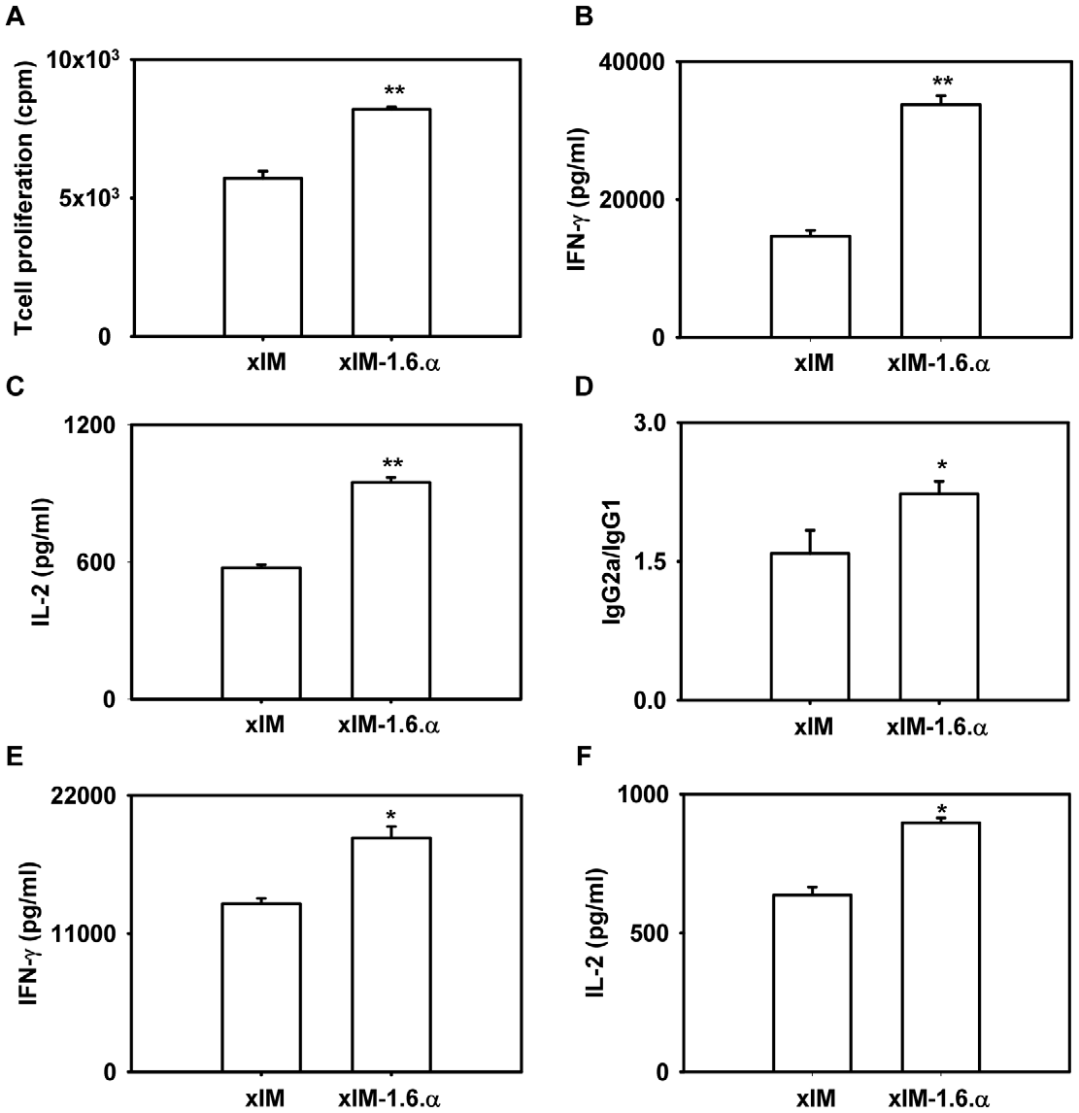
Vaccination with xIM-1.6.α evokes immune response. Mice were vaccinated with xIM-1.6.α with similar immunization protocol as mentioned in Figure 1. Lymphocytes pooled from spleen and lymphnodes of immunized animals were stimulated *in vitro* with PPD (50 µg/ml) for 48 h and later T cell proliferation was monitored by ^3^H-thymidine incorporation (A); secretion of IFN-γ (B) and IL-2 (C) in culture SNs; PPD specific IgG2a/IgG1 antibodies in the serum (1000× dilution) (D) were estimated by ELISA. Production of IFN-γ (E) and IL-2 (F) was estimated in the culture SNs of the lymphocytes isolated from lungs, *in vitro* stimulated with PPD (50 µg/ml). Data shown as mean ± SEM are representative of two experiments, n = 3 animals per group. ‘*’ and ‘**’ indicate p<0.05 and p<0.01 respectively.

**Figure 5 pone-0016097-g005:**
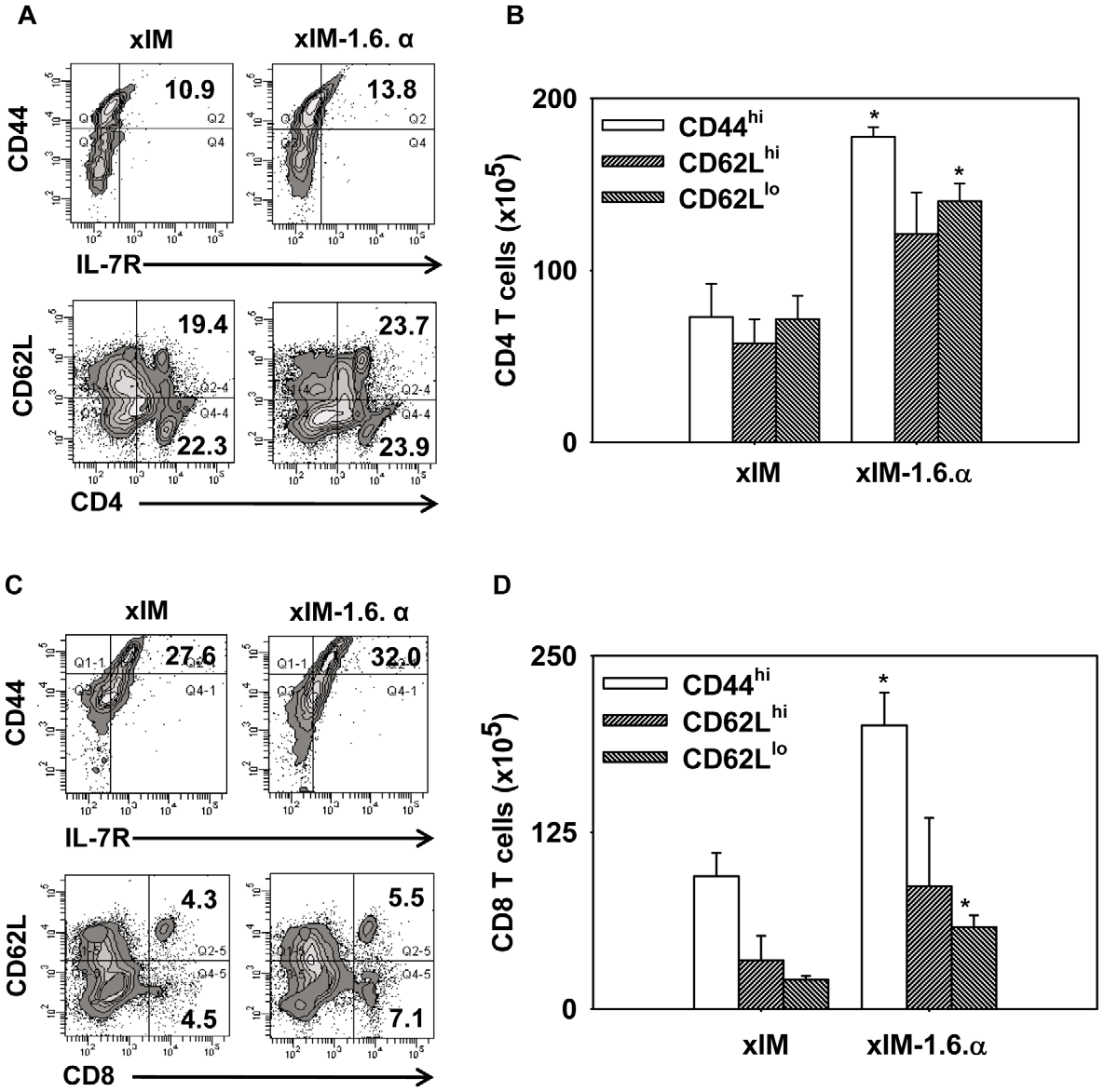
Vaccination with xIM-1.6.α induces CD4 and CD8 T cells memory response. Mice were immunized and aerosol challenged as mentioned in Figure 1. Pooled lymphocytes from spleen and lymphnodes of immunized animals were stimulated *in vitro* with PPD (50 µg/ml) for 48 h and later stained with fluorochrome tagged antibodies and analyzed by flowcytometry. A, flow contours depict percentage of CD4 T cells expressing IL-7R and CD44 (upper panel); CD62L expression on CD4 T cells (lower panel). C, CD8 T cells expressing IL-7R and CD44 (upper panel); CD62L on CD8 T cells (lower panel). Bar diagrams show total number of CD4 (B) and CD8 (D) T cells expressing CD44^hi^, CD62L^hi^ and CD62L^lo^. Data shown are mean ± SEM of two independent experiments with n = 3 animals per group. ‘*’ indicates p<0.05.

The requirement of syngeneic macrophages is a major concern in extrapolating mice data for practical human application. We showed earlier that vaccination with syngeneic, allogeneic and xenogeneic macrophages infected with *M. tb and S. typhimurium* or pulsed with ovalbumin could successfully mount an immune response against the entrapped antigens [5], [25]. Therefore we next ascertained whether IL-1+IL-6+TNF-α with infected xenogeneic (THP-1) macrophages (xIM-1.6.α) could provide similar outcome as observed in the case of syngeneic infected macrophages (sIM-1.6.α) (Figure 1, 2, 3). The cells isolated from spleen and lymph nodes of the immunized mice were *in vitro* stimulated with PPD. As observed in the case of sIM-1.6.α, similar pattern of enhancement of T cell proliferation (p = 0.004), IFN-γ (p = 0.0066), IL-2 (p = 0.0046) and IgG2a (p = 0.0317) secretion than control xIM alone was observed with xIM-1.6.α (Figure 4A–D). Further, like lymphoid organs, lymphocytes isolated from lungs also showed significantly better yield of IFN-γ (p = 0.0355) and IL-2 (p = 0.0164) (Figure 4E, F). Furthermore, no difference was observed in the production of IL-4 and IL-5 (Figure S6). The results were reproduced with all the tested antigenic preparations (PPD, STCF-H37Rv) (Figure 4, Figure S5). Furthermore, like sIM-1.6.α, augmentation in both central (CD44^hi^ CD62L^hi^) and effector (CD44^hi^ CD62L^lo^) memory CD4 and CD8 T cells was noticed on vaccination with xIM-1.6.α (Figure 5). Enhancement in both percentage (Figure 5A, C) and total number of cells (Figure 5B, D) was observed.

**Figure 6:**
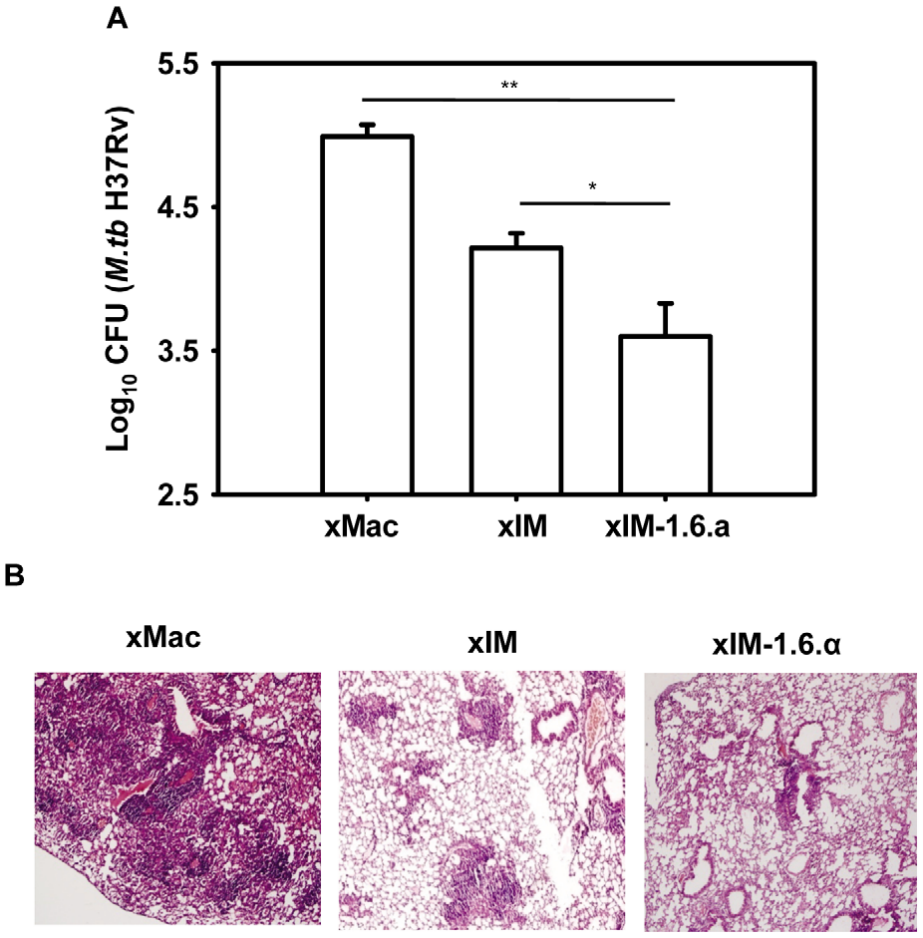
Immunization with xIM-1.6.α provides protection against *M. tb*. Mice vaccinated with xIM-1.6.α were rested for 240 days and then aerosol challenged with *M. tb*. After 35 days, mice were sacrificed and mycobacterial load was enumerated by CFU plating of diluted lung homogenates. A, data are represented as log_10_ CFU (n = 4–6 animals/group). B, photomicrographs (10×) of H & E stained lungs sections. Control groups of mice were inoculated with uninfected xenogeneic macrophages (xMac). Data are mean ± SEM of two independent experiments, where ‘*’ and ‘**’ indicate p<0.05 and p<0.01 respectively.

Lastly, we also checked the protective efficacy of xIM-1.6.α against *M. tb.* Mice vaccinated with xIM-1.6.α showed significantly better reduction in mycobacterial load in the lungs compared to uninfected control xenogeneic macrophages (p = 0.0015) and xIM (p = 0.0495) (Figure 6A) and its protective efficacy of was found to be similar with that of sIM-1.6.α (Figure S7). Further, alleviated lung pathology was observed (Figure 6B) and these results were comparable with sIM-1.6.α vaccine (Figure 3A, B and Figure 6A, B). These data very categorically establish that syngeneic macrophage is not a pre-requisite for this vaccination strategy, rather both syngeneic and xenogeneic macrophages can be employed with equal efficiently.

### Mechanism of priming of immune system by the vaccine

**Figure 7:**
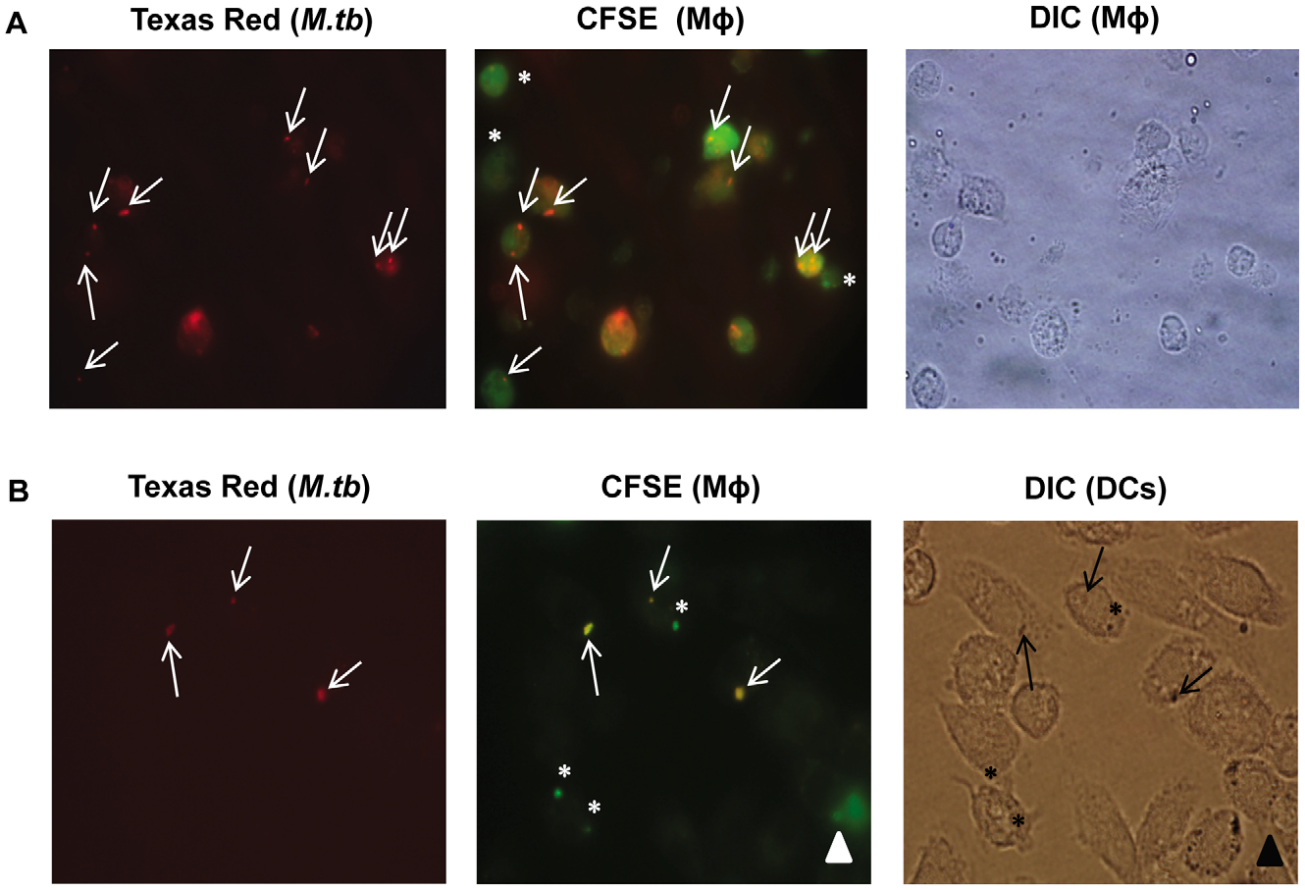
Apoptotic bodies generated by IM transport antigen to bystander dendritic cells. *M. tb* infected macrophages were co-cultured with Dendritic cells (DCs) for 48 h. Later, fluorescence microscopy was used to demonstrate generation of apoptotic bodies containing mycobacterial antigens and their uptake by bystander DCs. (A) Left panel, arrows indicate Texas Red labeled *M. tb*; Middle panel, *M. tb* infected macrophages (orange, mixed fluorescence) and asterisks depict uninfected macrophages (green); right panel, DIC image of macrophages (images at 100×). (B) Left panel, arrows indicate Texas Red labeled *M. tb*; middle panel, arrows indicate *M. tb* within apoptotic bodies (orange, mixed fluorescence) while asterisks indicate apoptotic bodies (green) devoid of mycobacteria; right panel, DIC image of DCs show engulfment of apoptotic bodies containing mycobacterial antigens by DCs (Images at 100×1.6×). Arrow heads indicate association of apoptotic macrophages with DCs. DIC, Differential interference contrast. () Data are representative of three independent experiments.

We hypothesize two possibilities which may explain generation of immune response by the vaccine. Firstly, *M. tb* infected syngeneic macrophages may directly present antigens to T cells. Secondly, infected macrophages (syngeneic and xenogeneic) on γ-irradiation may undergo apoptosis [5], [26] and resulting apoptotic vesicles contain processed mycobacterial antigens [27], [28]. These apoptotic vesicles can be avidly taken up by Dendritic cells (DCs), which then process and present antigens to CD4 T cells through MHC-II or cross present to CD8 T cells via MHC-I pathway [27], [28]. Therefore to demonstrate the antigenic transfer by apoptotic bodies to bystander DCs, Carboxyfluorescein diacetate succinimidyl ester (CFSE)-labeled macrophages were infected with Texas Red labeled *M. tb* (Figure 7A), followed by γ-irradiation to induce apoptosis [26]. This infected macrophage preparation was *in vitro* co-cultured with bone marrow derived dendritic cells (BMDCs). Interestingly, we observed the presence of apoptotic vesicles containing mycobacterial antigens (orange fluorescence) in DCs (Figure 7B), demonstrating that *M. tb* antigens from infected macrophages can get transported to DCs. These DCs can then process and present antigens to T cells and thereby imparting optimum protection by eliciting immune response (Figure 1–6).

## Discussion

Recently, cell based vaccines have gained considerable impetus following their success against cancer [11]. We had earlier demonstrated a novel strategy of vaccination by utilizing infected macrophages [5]. This vaccination approach offered significant protection not only against *M. tb* but also *S. typhimurium* [5]. However, it failed to induce long-lasting protection against *M. tb*. One of the cardinal features of a successful vaccine is its ability to elicit long-lasting memory. It is worth to mention here that even though BCG successfully protects against the infant TB, it fails in preventing the adult manifestation of the disease, reflecting poor induction of long-lasting T cell memory [29]. Over the recent years, studies have demonstrated a non-redundant role of cytokines in differentiation and maintenance of memory T cells [12], [13]. Common γ-chain and proinflammatory cytokines are known to induce and support the persistent T cell memory [12], [14]. These cytokines act directly on both naive and memory T cells and help in their survival and expansion [30]. Further, they also assist in the recruitment of antigen presenting cells at the site of vaccine administration.

Hence, in the light of above-mentioned facts, the present study was conducted. We evaluated the role of co-administration of common γ-chain (IL-7+IL-15) or proinflammatory (IL-1+IL-6+TNF-α) cytokines, with a cell based vaccine prepared using macrophages infected with *M. tb*, in generating enduring T cell memory and protection against TB. Mice were challenged with *M. tb* after 240 days of vaccination to ensure the legitimate long-term memory T cell responses [18]. The following major findings have emerged from this study: IM-1.6.α significantly (i) inducted a robust immune response that resulted in enduring memory CD4 and CD8 T cells; (ii) elicited a predominant Th1 immune response; (iii) reduced mycobacterial load; (v) alleviated the lung pathology; (vi) protected better than conventional BCG vaccine.

More than 90%–95% of the 2 billion individuals exposed to *M. tb* never develop active disease [2], indicating that the bacillus inhabiting the macrophages secretes unique molecules that stimulate the immune system for efficient control of the disease. Keeping this fact in the mind, we prepared a vaccine by infecting macrophages with live *M. tb* simulating a natural scenario. The preparation was made safe by killing the bacteria with isoniazid and γ-irradiated to induce apoptosis [5], [26]. Interestingly, incorporation of IL-1+IL-6+TNF-α with the vaccine significantly enhanced enduring T cell memory response.

Although this approach effectively protected the immunized mice from TB, the concern was its applicability to humans. Preparing syngeneic macrophage vaccines for humans may not be feasible. We had earlier demonstrated that major histocompatibility complex (MHC)–mismatched macrophages infected with mycobacterium can also be used successfully for vaccination [5], [25]. Interestingly, co-administration of IL-1+IL-6+TNF-α with xenogeneic vaccine (xIM-1.6.α) also elicited long-term immunity against *M. tb* and response was comparable to sIM-1.6.α (Figure S7).

We have also demonstrated the proof of principle of how this vaccine may eventually prime the immune system. Apoptotic bodies are likely to be generated by the infected macrophage vaccine. Such apoptotic bodies could be avidly taken up *in vivo* by DCs. Interestingly, we could locate apoptotic bodies loaded with mycobacterial antigens inside the DCs (Figure 7). Further, DCs but not macrophages can cross-present antigen of apoptotic cells to T cells because they express αvβ5 integrin, which plays a critical role in the trafficking of exogenous antigens [31], [32]. Hence, DCs that have phagocytosed apoptotic bodies containing mycobacterial antigens are likely to prime both naïve CD4 T cells (*via* exogenous pathway) and CD8 T cells (*via* cross presentation) to generate protective immune response against *M. tb*. Furthermore, as DCs secrete IL-12, they are capable of skewing immune response towards IFN-γ secreting CD4 and CD8 T cells [33]. The proinflammatory stimuli is also necessary for diverting the immune response towards protective Th17 cells, which is reported to be important for the rapid recruitment of Th1 effectors to the lungs [34], [35]. Moreover, DCs trap foreign antigen (in this case mycobacterium antigen) and act as reservoir, slowly releasing the antigen in the system for the activation of T cells and maintenance of memory cells [36]. Hence, incorporating IL-1+IL-6+TNF-α with vaccine, promotes the generation of long-term memory T cells.

Based on our findings, we envisage that the strategy of utilizing MHC-mismatched macrophages can work as a promiscuous vaccine for humans. Importantly, the preparation requires no adjuvant and shows better protection than BCG. Therefore, we conclude that IM-1.6.α may be a possible vaccine candidate against TB, based on its uniqueness of utilizing novel antigens (secreted within the natural environment of the macrophages which may be of paramount importance for evoking protection) and presence of IL-1+IL-6+TNF-α (for induction and sustenance of enduring CD4 and CD8 T cell memory responses).

## Materials and Methods

### Experimental animals

BALB/c mice (female, 6–8 weeks old) were procured from National Institute of Pharmacological Education and Research, Mohali, India and National Institute of Immunology, New Delhi, India. Animals were kept in Biosafety Level-3 facility of Institute of Microbial Technology (IMT), Chandigarh, India and National JALMA Institute for Leprosy and Other Mycobacterial Diseases (NJIL&OMD), Agra, India. The animals were offered pellet feed and water *ad libitum*.

### Ethics Statement

All animal experiments were approved by the Institutional Animal Ethics Committees of IMT and NJIL&OMD. All animal experiments were performed according to the National Regulatory Guidelines issued by CPSEA (Committee for the Purpose of Supervision of Experiments on Animals), Ministry of Environment and Forest, Govt. of India.

### Reagents and antibodies

Middlebrook 7H9, 7H11, ADC and OADC were procured from Difco Laboratories (Sparks, MD). Recombinant mouse IL-1, IL-6, TNF-α, IL-7 and IL-15 from Peprotech (Rocky Hill, NJ). Fluorochrome labeled anti-mouse antibodies, FITC-conjugated CD4 (H129.19), IgG2a isotype control (R35-95); PE-conjugated CD62L (MEL-14), IgG2b isotype control (R35-95), IgG2c isotype control (A23-1); PE-Cy5-conjugated CD44 (IM7), Sv-PE-Cy5; Pacific Blue conjugated CD4 and IgG2a isotype control (R35-95); APC-Cy7-conjugated CD8 (53-6.7) and IgG2a isotype control (R35-95); biotin-conjugated CD4 (H129.19) and IgG2a isotype control (R35-95) and pair matched antibodies for ELISA, JES6-1A12, JES6-5H4 (IL-2); 11B11, BVD6-24G2 (IL-4); TRFK5, TRFK4 (IL-5); R4-6A2, XMG1.2 (IFN-γ), A85-1 (IgG1-Biotin), R19-15 (IgG2a-biotin) procured from BD Biosciences (San Diego, CA). FITC-conjugated CD8 (KT15) and IgG2a isotype control were from AbD Serotec (Langford Lane, Kidlington, UK). Tissue culture grade plastic-wares were purchased from BD Biosciences (Bedford, MA) and Nunc (Rochester, NY). Texas Red succinimidyl ester was procured from Invitrogen (Eugene, OR). All other standard reagents were purchased from Sigma (St. Louis, MO) unless mentioned.

### Mycobacterial strain and antigens

*M. tb* H37Rv and *M. bovis* BCG (Danish strain) were kind gifts from Dr. V. M. Katoch, NJIL&OMD, Agra. Mycobacteria were cultured in 7H9 medium containing 0.05% Tween80 supplemented with 10% ADC enrichment. Short term culture filtrates (STCF) and purified protein derivative (PPD) were prepared by standard protocols [19]. Briefly, the mycobacteria from log phage cultures were grown in 7H9 broth without ADC enrichment and Tween80 for 2 weeks at 37°C. The culture supernatant was harvested by centrifugation and filtered through 0.2 µm membrane filter. Supernatant containing culture filtrate proteins were lyophilized. Lyophilized proteins were dissolved and dialyzed extensively against PBS and stored at −80°C for future use. For PPD preparation, cultures were incubated for 8–9 weeks at 37°C. Later, cultures were autoclaved at 121°C for 30 min and cooled overnight at room temperature. Bacterial cells were removed by centrifugation followed by filtration. The proteins in the filtrate were precipitated by trichloroacetic acid (4% final concentration) by keeping overnight at room temperature without any agitation. Later, the precipitate was centrifuged at 2500×*g* for 15 min to remove supernatant. The precipitate was washed 2× with 1% TCA and once with 10% NaCl. Then precipitate was dissolved in PBS, filter sterilized and stored at −80°C for future use.

### Cells and cell lines

#### Peritoneal macrophages

Mice were inoculated with 3 ml of 3% thioglycollate medium intraperitoneally. After 96 h, peritoneal exudates cells (PECs) were harvested and washed 2× with RPMI-1640-FBS-1%. Macrophages were purified by adhering on 90 mm tissue culture dish for 2 h.

#### THP-1

A human macrophage cell line was maintained in RPMI-1640-FBS-5% and used as source of xenogeneic macrophages for vaccine preparation.

#### Bone marrow derived dendritic cells (BMDC)

Bone marrow cells were isolated from femur of BALB/c mice and cultured (2×10^6^) in 60 mm Petri dish in the presence of GM-CSF (10 ng/ml) and IL-4 (5 ng/ml) in complete RPMI-1640-FBS-10% for 6 days. Cultures were replenished with fresh GM-CSF and IL-4 after 3 days. Cells were harvested on day 7 for experiments.

### Preparation of infected macrophages and cytosolic antigenic proteins

Infected syngeneic (sIM) and xenogeneic (xIM) macrophage vaccines for immunization and infected macrophages cytosolic (IMC) antigenic proteins for *in vitro* studies were prepared by culturing live *M. tb* inside the macrophages as described earlier [5]. Briefly, syngeneic peritoneal macrophages and xenogeneic THP-1 cells were plated overnight at 37°C in antibiotic free RPMI 1640-FBS-5%. The cells were then infected with live *M. tb* (MOI, 1∶20) for 4 h. Extracellular bacteria were removed by extensive washing with PBS. Infected cells were cultured with amikacin (50 µg/ml) for 48 h to check residual extracellular bacteria, if any. Isoniazid (100 µg/ml) treatment was given for next 48 h to kill intracellular bacteria. The cells were harvested and γ-irradiated (5000 rad). The cells were washed 2× and resuspended at a concentration of 5×10^6^ cells/ml in PBS. For preparation of IMC, infected macrophages were lysed in a buffer containing 10 nmol/L Tris (pH 8.0), 1% Triton X-100, 150 nmol/L NaCl, 5 mmol/L EDTA, 1 mmol PMSF, 10 mg/L aprotinin and 10 mg/L leupeptin. Disrupted cells were centrifuged at 600×g for 20 min, supernatant was collected and ultracentrifuged at 100,000×g for 1 h at 4°C. Supernatant was then dialyzed extensively against PBS and stored at −80°C for future use.

### Immunization

Syngeneic (sIM) or xenogeneic (xIM) infected macrophages vaccine (5×10^5^ macrophages/100 µl PBS/mouse) alone or along with recombinant IL-1, IL-6 (500 ng/mouse, each cytokine) and TNF-α (250 ng/mouse) (IM-1.6.α) or IL-7 and IL-15 (500 ng/mouse, each cytokine) (IM-7.15) were injected intraperitoneally. After 21 days, booster dose of same preparation was given. Control mice were administered with placebo (PBS or 1.6.α alone), BCG and BCG-1.6.α. To establish a *bona fide* memory response, animals were rested for 240 days before aerosol challenge [18].

### Aerosol infection and determination of mycobacterium burden

Mice were infected with a low dose aerosol challenge with *M. tb* to deposit approximately 100 bacteria/lung using an inhalation exposure system (Glas-Col, Terre Haute, IN) [19]. Briefly, animals were exposed to *M. tb* aerosol for 45 min with a standardized dose. Numbers of mycobacteria deposited were determined by plating total lung homogenates on Middlebrook 7H11 plates supplemented with OADC enrichment within 24 h. The mycobacterium burden in the immunized animals was determined after 35 days of aerosol challenge by plating serially diluted homogenates of individual lung. CFUs were counted after 3–4 weeks of incubation at 37°C and total load of lung tissue was expressed as log_10_ CFU.

### Isolation of lymphocytes from spleen, lymph nodes and lungs

Mice were sacrificed after 35 days of aerosol challenge with *M. tb*. A single cell suspension from spleen, lymph nodes (inguinal, axillary and brachial) and lungs was prepared as described earlier [19]. Briefly, lymphocytes from spleen were prepared by RBCs lysis with ACK (NH_4_Cl 0.15 M, KHCO_3_ 10 mM and EDTA 88 µM) buffer, washed 3× with PBS and resuspended in RPMI-1640-FBS-10%. For lymphocytes from lungs, perfused lungs were minced and digested with collagenase (0.7 mg/ml) and DNase (30 µg/ml) at 37°C for 1 h. Digested lungs were disrupted gently by passage through a 70-µm pore size cell strainers; residual RBCs, if any, were lysed by ACK buffer. The resultant single cell suspension was washed 3× and resuspended in RPMI-1640-FBS-10%. Viable cells were counted by trypan blue dye exclusion method.

### Proliferation assays

Lymphoproliferation assays were set as described previously [5], [19]. Briefly, lymphocytes isolated from lymphoid (spleen and lymph nodes) and non-lymphoid (perfused lungs) organs of mice (2×10^5^ cells/well) were cultured in round-bottom 96-well plates, in 200 µl of RPMI-1640-FBS-10% and different concentrations (1.56–50 µg/ml) of mycobacterial antigens (PPD, STCF-H37Rv, IMC) for 48 h and 72 h respectively. Cells cultured with the medium alone (without antigens) were used as controls. Later, cultures were pulsed with 0.5 µCi of ^3^H-thymidine (Amersham Pharmacia Biotech, Buckinghamshire, UK). The plates were harvested after 16 h onto glass-fiber filter mats using a Tomtec-Harvester-96 (Tomtec, Hamden, CT). Radioactivity incorporated was measured using liquid scintillation spectroscopy by Wallac-1450 Microbeta Trilux (Perkin Elmer, Waltham, MA).

### Cytokines ELISA

Cultures were set as described in case of proliferation assays. The culture supernatants (SNs) from the experimental and control wells were collected after 24 h for IL-2 and 48 h for IL-4, IL-5 and IFN-γ estimation. Cytokines were estimated by sandwich ELISA following manufacturer's instructions [5], [19]. The levels of cytokines were calculated using standard recombinant cytokines and expressed as pg/ml.

### IgG isotypes ELISA

Serum samples were collected from clotted blood after 35 days of aerosol challenge. *M. tb* specific antibodies were determined in the serum of vaccinated mice, as described elsewhere [5], [19]. Briefly, diluted serum samples (1000×) were added on PPD (10 µg/ml) coated plates. PPD specific antibodies were captured by secondary biotinylated anti-mouse IgG1/IgG2a followed by avidin-HRP/OPD-H_2_O_2_ step for colorimetric estimation. Usual steps of washings and incubations were followed at each step. Results were expressed as ratio of IgG2a to IgG1.

### Staining for T cell memory surface markers

Cells were stimulated with antigens as described for proliferation assays. After 48 h of cultures, cells were stained for surface memory markers[19]. Briefly, cells were harvested and washed 2× with staining buffer (PBS-FBS-1%). Cells were incubated with 2.4G2 antibodies to block Fc-receptors and stained with anti-mouse fluorochrome labeled monoclonal antibodies (mAbs) (CD4, CD8, CD44, CD62L, CD127 and isotype controls) for 30 min at 4°C. When staining with biotinylated antibodies, cells were incubated with biotin conjugated mAbs for 30 min at 4°C followed by incubation with secondary reagents (streptavidin-PE-Cy5 or APC). Usual steps of washing and incubation were followed at each interval. Finally, cells were fixed in 1% paraformaldehyde. The cytometry data were acquired using FACS Calibur and FACS Aria II (BD Biosciences, San Jose, CA). Data were analyzed by DIVA software (BD Biosciences, San Jose, CA). Total number of cells of a definite phenotype of CD4^+^ and CD8^+^ T cells (CD44^hi^, CD62L^hi/lo^) was calculated by taking the percentage of gated cell type, as determined by flowcytometry, multiplied by the total number of cells [22].

### Histo-pathological analysis

Mice were sacrificed and lung tissues were fixed in 10% buffered formalin. Histological sections were stained using hematoxylin and eosin. Photomicrographs were captured on Olympus IX71 microscope.

### Engulfment of apoptotic bodies by Dendritic cells

Mycobacteria were stained with Texas Red as per manufacturer's instructions. Briefly, single cell suspension of *M. tb* were incubated with Texas Red (50 µg/ml) at room temperature for 2 h followed by washing 5× to remove excess of stain. Peritoneal exudate cells (PECs) were incubated with 3 µM of Carboxyfluorescein diacetate succinimidyl ester (CFSE) in PBS for 8 min at 37°C. Excess of CFSE was removed by treating with 100% FBS and followed by washing 3× with RPMI-FBS-10%. CFSE labeled macrophages were purified by adhering on 90 mm tissue culture dish for 2 h. Non adhered cells were removed by washings with PBS. Adhered macrophages were infected with Texas Red stained *M. tb* (MOI, 1∶20) for 4 h. Extracellular bacteria were removed by extensive washings with PBS. Infected macrophages were harvested and washed 2× with PBS-FBS-1% and subjected to γ-irradiation to induce apoptosis [26]. BMDCs (2×10^5^ cells/200 µl) were adhered on glass cover slips for 4 h, followed by 3× washing to remove unattached cells. Gamma irradiated *M. tb* infected macrophages were co-cultured with BMDCs on glass cover slips in 1∶1 ratio for 48 h. Later, cover slips were washed 5× with PBS and fixed with 4% paraformaldehyde. Fluorescence images were captured on Olympus IX71 microscope.

### Statistical analysis

Data were analyzed by one way analysis of variance (ANOVA) with post Tukey-Kramer multiple comparisons test. In case of significant difference, absolute ‘p’ values for comparison between two groups were calculated by Student's‘t’ test using InStat 3 software (GraphPad).

## Supporting Information

Figure S1 **sIM-1.6.α exhibits a long-lasting Th1 immune response on** ***in vitro*** **stimulation with STCF-H37Rv.** Mice were vaccinated with sIM-1.6.α. After 240 days, they were aerosol challenged with *M. tb* and 35 days later were sacrificed. Lymphocytes Pooled from spleen and lymphnodes (A, B, C) and lungs (D, E) of immunized mice were stimulated *in vitro* with STCF-H37Rv (50 µg/ml). T cell proliferation was monitored by ^3^H-thymidine incorporation (A); secretion of IFN-γ (B, D) and IL-2 (C, E) by ELISA in the culture SNs. The control groups were administered with PBS, BCG and sIM. Data are shown as mean ± SEM and representative of two experiments, n = 3 animals per group. ‘*’, ‘**’and ‘***’ indicate p<0.05, p<0.01 and p<0.001 respectively.
(TIF)

Figure S2 **sIM-1.6.α demonstrates a long-lasting Th1 immune response against IMC.** Mice were vaccinated as mentioned in Figure S1. Lymphocytes Pooled from spleens and lymph nodes (B, C) and lungs (D, E) were stimulated *in vitro* with IMC (50 µg/ml). T cell proliferation was monitored by ^3^H-thymidine incorporation (A); production of IFN-γ (B, D) and IL-2 (C, E) in the culture SNs by ELISA. The control groups were administered with PBS, BCG and sIM. Data are shown as mean ± SEM and representative of two experiments, n = 3 animals per group. ‘*’ and ‘**’ indicate p<0.05 and p<0.01 respectively.
(TIF)

Figure S3 **sIM-1.6.α does not increase Th2 response.** Mice were vaccinated as mentioned in Figure S1. Lymphocytes Pooled from spleens and lymph nodes (A) and lungs (B) were stimulated *in vitro* with PPD, STCF-H37Rv and IMC (50 µg/ml). Secretion of IL-4 and IL-5 was monitored in the culture SNs. The control groups were administered with PBS, BCG and sIM. Data are shown as mean ± SEM and representative of two experiments, n = 3 animals per group.
(TIF)

Figure S4 **sIM-7.15 and BCG-1.6.α failed to show any protection.** sIM-7.15 and BCG-1.6.α vaccinated mice were rested for 240 days before aerosol challenge. After 35 days, animals were sacrificed; mycobacterial load was enumerated by CFU plating. Control groups were immunized with syngeneic uninfected macrophages (sMac), BCG and cytokines alone (1.6.α and 7.15). Data are represented as mean ± SEM of log_10_ CFU (n = 4–5 animals/group) of two independent experiments.
(TIF)

Figure S5 **Immunization with xIM-1.6.α also elicits robust immune response against STCF-H37Rv.** Mice were vaccinated with xIM-1.6.α and xIM. After 240 days, they were aerosol challenged with *M. tb* and 35 days later were sacrificed. Lymphocytes Pooled from spleens and lymph nodes (A, B, C) and lungs (D, E) were stimulated *in vitro* with STCF-H37Rv (50 µg/ml). T cell proliferation was monitored by ^3^H-thymidine incorporation (A); release of IFN-γ (B, D) and IL-2 (C, E) in the culture SNs by ELISA. Data are shown as mean ± SEM and representative of two experiments, n = 3 animals per group. ‘*’ and ‘**’ indicate p<0.05 and p<0.01 respectively.
(TIF)

Figure S6 **xIM-1.6.α does not augment Th2 response.** Mice were vaccinated as mentioned in Figure S5. Lymphocytes pooled from spleens and lymph nodes (A) and lungs (B) were stimulated *in vitro* with PPD and STCF-H37Rv (50 µg/ml). Secretion of IL-4 and IL-5 was estimated in the culture SNs. Data are shown as mean ± SEM and representative of two experiments, n = 3 animals per group.
(TIF)

Figure S7 **sIM-1.6.α and xIM-1.6.α provides comparable protection against** ***M. tb*** **.** Mice were vaccinated with sIM-1.6.α and xIM-1.6.α and rested for 240 days before aerosol challenge with *M. tb*. After 35 days, mice were sacrificed and mycobacterial load was enumerated by CFU plating of diluted lung homogenates. Control groups were inoculated with PBS and BCG. Data are represented as mean ± SEM of log_10_ CFU (n = 4–5 animals/group) of two independent experiments. ‘ns’, ‘*’, ‘**’and ‘***’ indicate non-significant, p<0.05, p<0.01 and p<0.001 respectively.
(TIF)

## References

[1] WHO (1992). Expanded program for immunization: program report..

[2] WHO (2009). Tuberculosis fact sheet number 104, World Health Organization..

[3] WHO (1998). Global tuberculosis control..

[4] Andersen P, Smedegaard B (2000). CD4(+) T-cell subsets that mediate immunological memory to Mycobacterium tuberculosis infection in mice.. Infect Immun.

[5] Sharma N, Agrewala JN (2004). Potent role of vaccines prepared from macrophages infected with live bacteria in protection against Mycobacterium tuberculosis and Salmonella typhimurium infections.. J Infect Dis.

[6] Orme IM, McMurray DN, Belisle JT (2001). Tuberculosis vaccine development: recent progress.. Trends Microbiol.

[7] Kaufmann SH, Baumann S, Nasser Eddine A (2006). Exploiting immunology and molecular genetics for rational vaccine design against tuberculosis.. Int J Tuberc Lung Dis.

[8] Orme IM (2005). Current progress in tuberculosis vaccine development.. Vaccine.

[9] Palucka AK, Ueno H, Fay JW, Banchereau J (2007). Taming cancer by inducing immunity via dendritic cells.. Immunol Rev.

[10] Moll H (2003). Dendritic cells as a tool to combat infectious diseases.. Immunol Lett.

[11] Ueno H, Schmitt N, Klechevsky E, Pedroza-Gonzalez A, Matsui T (2010). Harnessing human dendritic cell subsets for medicine.. Immunol Rev.

[12] Dooms H, Abbas AK (2006). Control of CD4+ T-cell memory by cytokines and costimulators.. Immunol Rev.

[13] Schluns KS, Lefrancois L (2003). Cytokine control of memory T-cell development and survival.. Nat Rev Immunol.

[14] Haynes L, Eaton SM, Burns EM, Rincon M, Swain SL (2004). Inflammatory cytokines overcome age-related defects in CD4 T cell responses in vivo.. J Immunol.

[15] Pape KA, Khoruts A, Mondino A, Jenkins MK (1997). Inflammatory cytokines enhance the in vivo clonal expansion and differentiation of antigen-activated CD4+ T cells.. J Immunol.

[16] Melchionda F, Fry TJ, Milliron MJ, McKirdy MA, Tagaya Y (2005). Adjuvant IL-7 or IL-15 overcomes immunodominance and improves survival of the CD8+ memory cell pool.. J Clin Invest.

[17] Rochman Y, Spolski R, Leonard WJ (2009). New insights into the regulation of T cells by gamma(c) family cytokines.. Nat Rev Immunol.

[18] Orme IM (2006). Preclinical testing of new vaccines for tuberculosis: a comprehensive review.. Vaccine.

[19] Singh V, Gowthaman U, Jain S, Parihar P, Banskar S (2010). Co-administration of IL-7 and IL-15 with BCG mount enduring T cell memory response against M. tuberculosis.. J Infect Dis.

[20] Jung YJ, Ryan L, LaCourse R, North RJ (2005). Properties and protective value of the secondary versus primary T helper type 1 response to airborne Mycobacterium tuberculosis infection in mice.. J Exp Med.

[21] Stevens TL, Bossie A, Sanders VM, Fernandez-Botran R, Coffman RL (1988). Regulation of antibody isotype secretion by subsets of antigen-specific helper T cells.. Nature.

[22] Wolf AJ, Desvignes L, Linas B, Banaiee N, Tamura T (2008). Initiation of the adaptive immune response to Mycobacterium tuberculosis depends on antigen production in the local lymph node, not the lungs.. J Exp Med.

[23] Swain SL, Agrewala JN, Brown DM, Jelley-Gibbs DM, Golech S (2006). CD4+ T-cell memory: generation and multi-faceted roles for CD4+ T cells in protective immunity to influenza.. Immunol Rev.

[24] Agrewala JN, Brown DM, Lepak NM, Duso D, Huston G (2007). Unique ability of activated CD4+ T cells but not rested effectors to migrate to non-lymphoid sites in the absence of inflammation.. J Biol Chem.

[25] Agrewala JN, Suvas S, Singh V, Vohra H (2003). Delivery of antigen in allogeneic cells preferentially generates CD(4+) Th1 cells.. Clin Exp Immunol.

[26] Hernandez-Flores G, Gomez-Contreras PC, Dominguez-Rodriguez JR, Lerma-Diaz JM, Ortiz-Lazareno PC (2005). Gamma-irradiation induced apoptosis in peritoneal macrophages by oxidative stress. Implications of antioxidants in caspase mitochondrial pathway.. Anticancer Res.

[27] Schaible UE, Winau F, Sieling PA, Fischer K, Collins HL (2003). Apoptosis facilitates antigen presentation to T lymphocytes through MHC-I and CD1 in tuberculosis.. Nat Med.

[28] Winau F, Weber S, Sad S, de Diego J, Hoops SL (2006). Apoptotic vesicles crossprime CD8 T cells and protect against tuberculosis.. Immunity.

[29] Fine PE (2001). BCG: the challenge continues.. Scand J Infect Dis.

[30] Ben-Sasson SZ, Hu-Li J, Quiel J, Cauchetaux S, Ratner M (2009). IL-1 acts directly on CD4 T cells to enhance their antigen-driven expansion and differentiation.. Proc Natl Acad Sci U S A.

[31] Albert ML, Sauter B, Bhardwaj N (1998). Dendritic cells acquire antigen from apoptotic cells and induce class I-restricted CTLs.. Nature.

[32] Rubartelli A, Poggi A, Zocchi MR (1997). The selective engulfment of apoptotic bodies by dendritic cells is mediated by the alpha(v)beta3 integrin and requires intracellular and extracellular calcium.. Eur J Immunol.

[33] Hickman SP, Chan J, Salgame P (2002). Mycobacterium tuberculosis induces differential cytokine production from dendritic cells and macrophages with divergent effects on naive T cell polarization.. J Immunol.

[34] Veldhoen M, Hocking RJ, Atkins CJ, Locksley RM, Stockinger B (2006). TGFbeta in the context of an inflammatory cytokine milieu supports de novo differentiation of IL-17-producing T cells.. Immunity.

[35] Khader SA, Bell GK, Pearl JE, Fountain JJ, Rangel-Moreno J (2007). IL-23 and IL-17 in the establishment of protective pulmonary CD4+ T cell responses after vaccination and during Mycobacterium tuberculosis challenge.. Nat Immunol.

[36] Kim TS, Hufford MM, Sun J, Fu YX, Braciale TJ (2010). Antigen persistence and the control of local T cell memory by migrant respiratory dendritic cells after acute virus infection.. J Exp Med.

