# Correction: Co-Administration of IL-1+IL-6+TNF-α with *Mycobacterium tuberculosis* Infected Macrophages Vaccine Induces Better Protective T Cell Memory than BCG

**DOI:** 10.1371/journal.pone.0092631

**Published:** 2014-03-18

**Authors:** The PLOS ONE Staff

Figure 3B is incorrect. The authors have provided a corrected version here.

**Figure 3.**
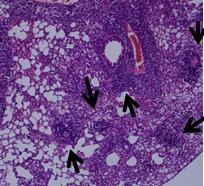
sIM-1.6.a provides significantly better protection than BCG. Mice were immunized and aerosol challenged as mentioned in Figure 1. Mycobacterial load in the lungs was enumerated by CFU plating of diluted lung homogenates. A, data are represented as log_10_ CFU (n  =  6 animals/group) where each triangle indicates single mouse. B, photomicrographs (106) of formalin fixed lungs sections stained with H & E. ‘+’ indicates active TB lesion with edematous follicular reaction while ‘R’ shows small or large developing follicular granulomas. Data are representative of two independent experiments. ‘**’ and ‘***’indicate p,0.01 and p,0.001 respectively.
