# Supplementary figures and images for: Co-Administration of IL-1+IL-6+TNF-α with Mycobacterium tuberculosis Infected Macrophages Vaccine Induces Better Protective T Cell Memory than BCG
Source: PLoS One. 2011 Jan 19;6(1):e16097. doi: 10.1371/journal.pone.0016097 (PMC3023717; doi:10.1371/journal.pone.0016097)

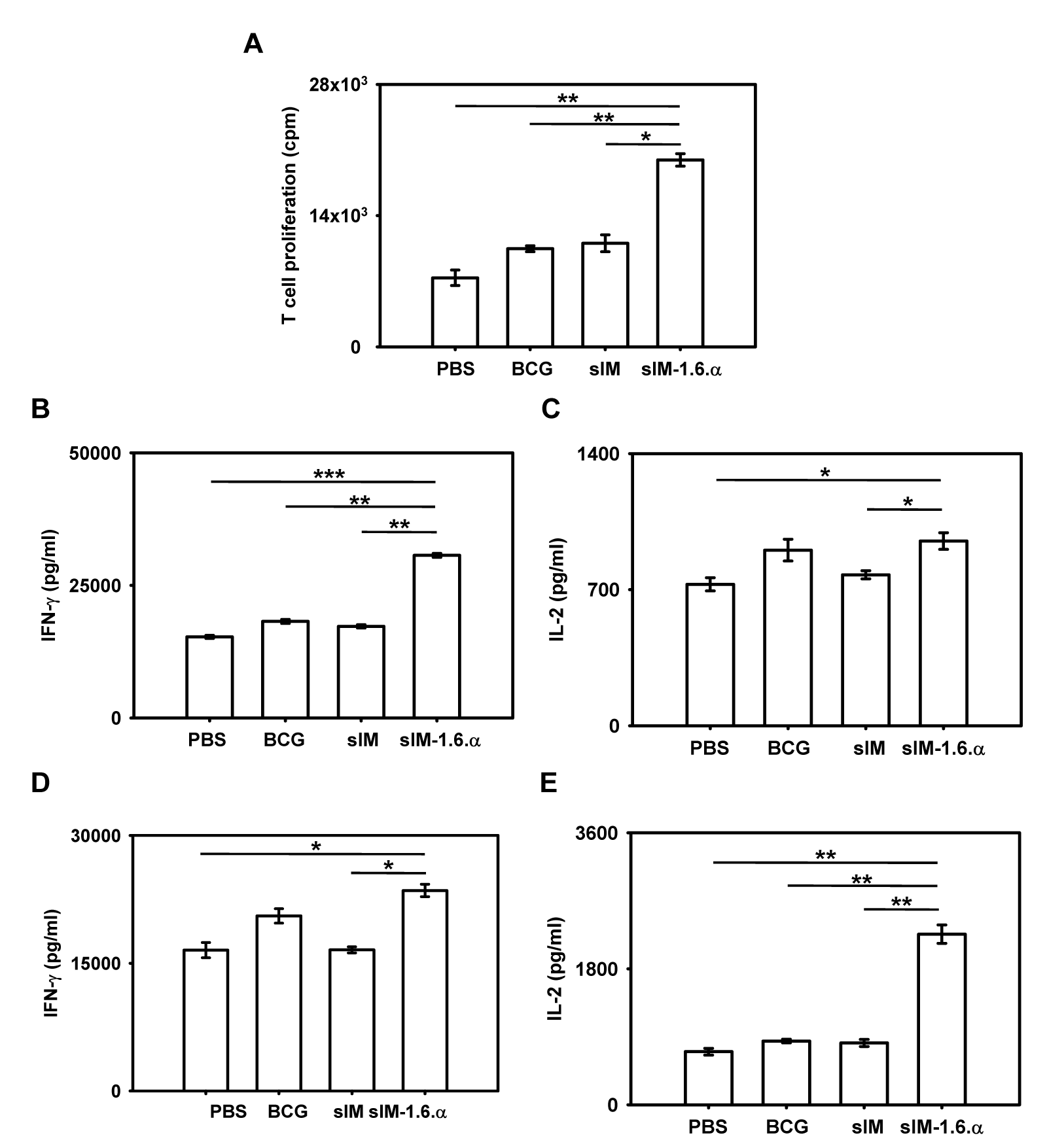

Supplement: Figure S1 — sIM-1.6.α exhibits a long-lasting Th1 immune response on in vitro stimulation with STCF-H37Rv. Mice were vaccinated with sIM-1.6.α. After 240 days, they were aerosol challenged with M. tb and 35 days later were sacrificed. Lymphocytes Pooled from spleen and lymphnodes (A, B, C) and lungs (D, E) of immunized mice were stimulated in vitro with STCF-H37Rv (50 µg/ml). T cell proliferation was monitored by 3H-thymidine incorporation (A); secretion of IFN-γ (B, D) and IL-2 (C, E) by ELISA in the culture SNs. The control groups were administered with PBS, BCG and sIM. Data are shown as mean ± SEM and representative of two experiments, n = 3 animals per group. ‘*’, ‘**’and ‘***’ indicate p<0.05, p<0.01 and p<0.001 respectively. (TIF) [file pone.0016097.s001.tif]

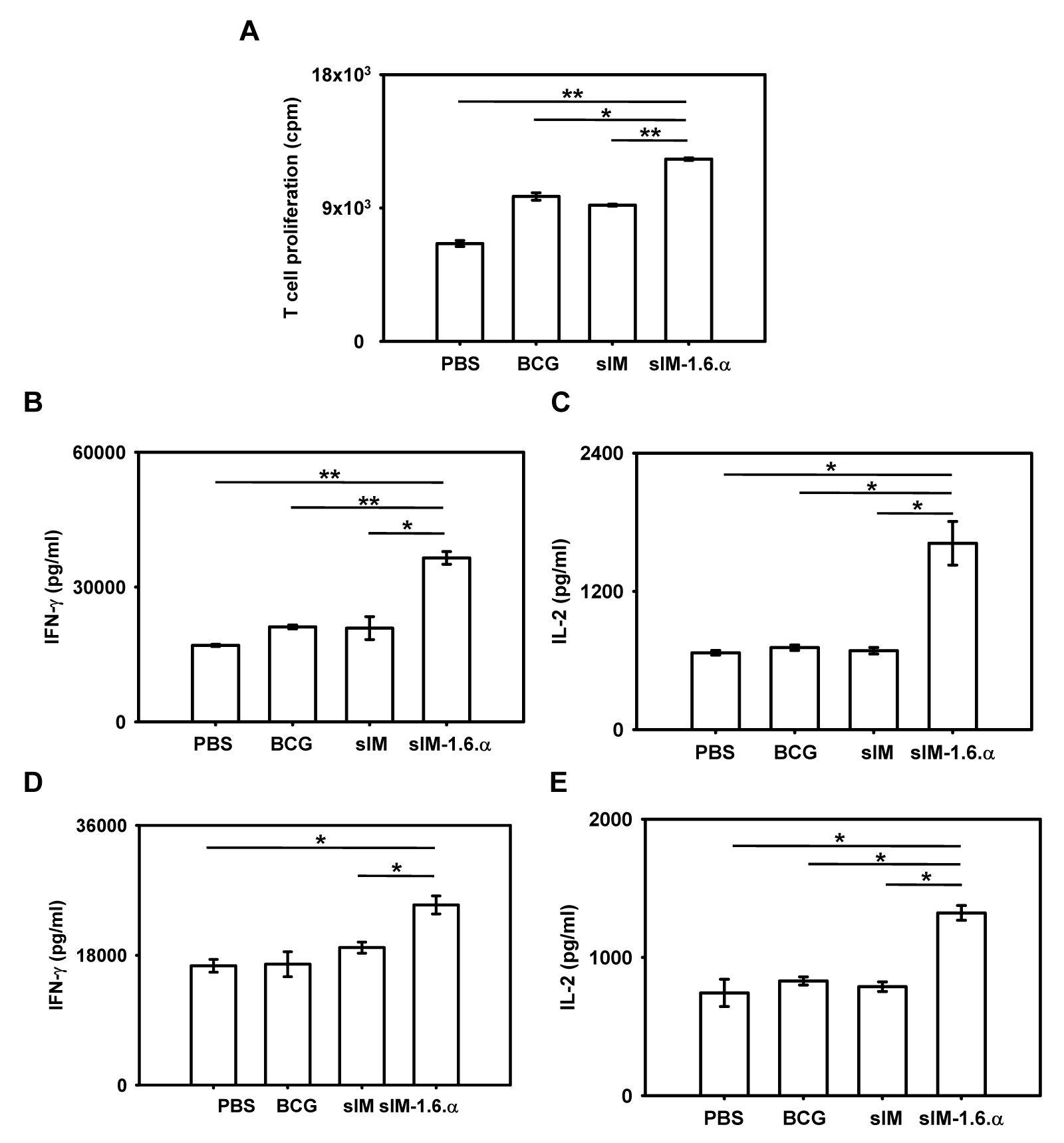

Supplement: Figure S2 — sIM-1.6.α demonstrates a long-lasting Th1 immune response against IMC. Mice were vaccinated as mentioned in Figure S1. Lymphocytes Pooled from spleens and lymph nodes (B, C) and lungs (D, E) were stimulated in vitro with IMC (50 µg/ml). T cell proliferation was monitored by 3H-thymidine incorporation (A); production of IFN-γ (B, D) and IL-2 (C, E) in the culture SNs by ELISA. The control groups were administered with PBS, BCG and sIM. Data are shown as mean ± SEM and representative of two experiments, n = 3 animals per group. ‘*’ and ‘**’ indicate p<0.05 and p<0.01 respectively. (TIF) [file pone.0016097.s002.tif]

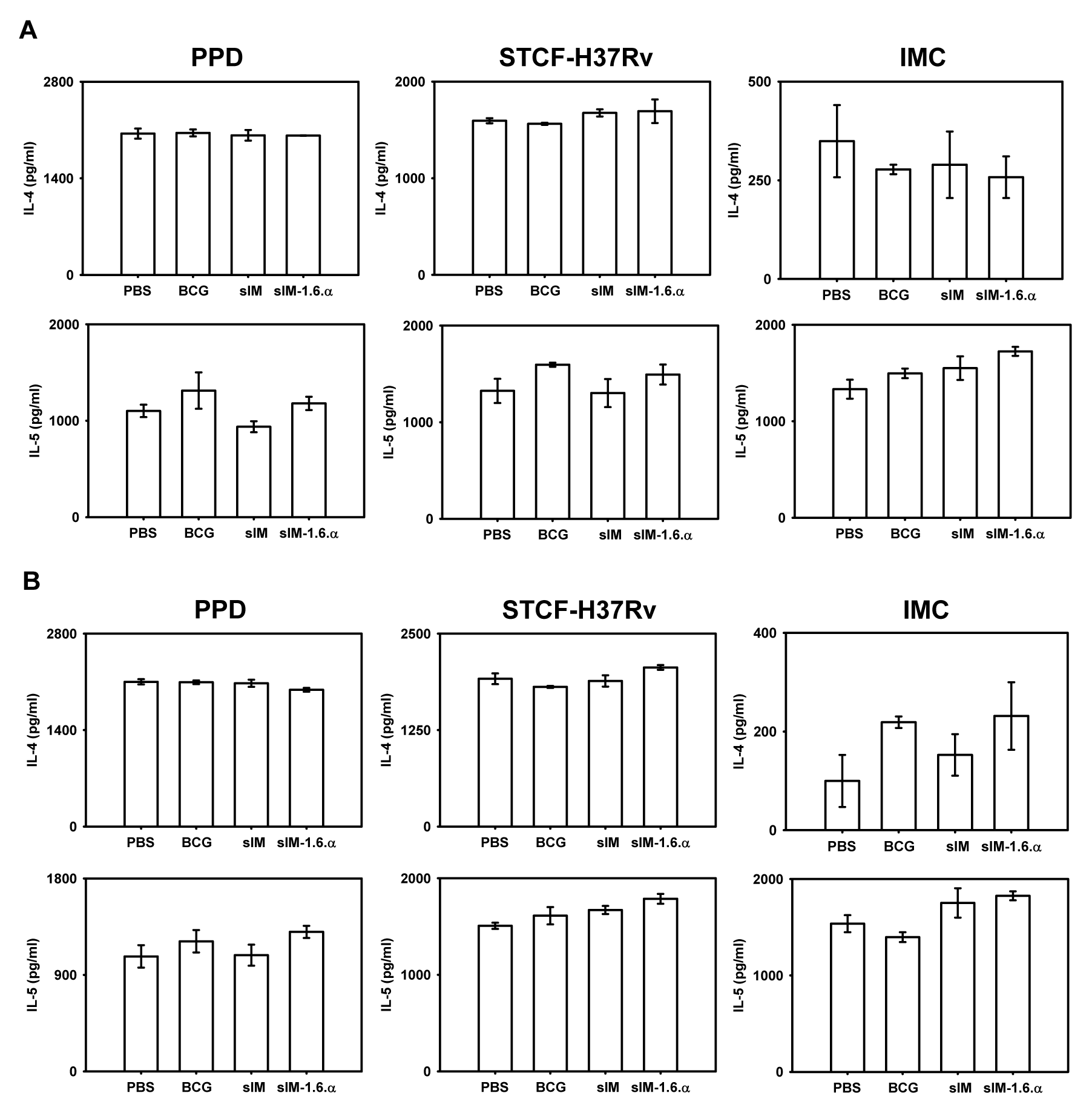

Supplement: Figure S3 — sIM-1.6.α does not increase Th2 response. Mice were vaccinated as mentioned in Figure S1. Lymphocytes Pooled from spleens and lymph nodes (A) and lungs (B) were stimulated in vitro with PPD, STCF-H37Rv and IMC (50 µg/ml). Secretion of IL-4 and IL-5 was monitored in the culture SNs. The control groups were administered with PBS, BCG and sIM. Data are shown as mean ± SEM and representative of two experiments, n = 3 animals per group. (TIF) [file pone.0016097.s003.tif]

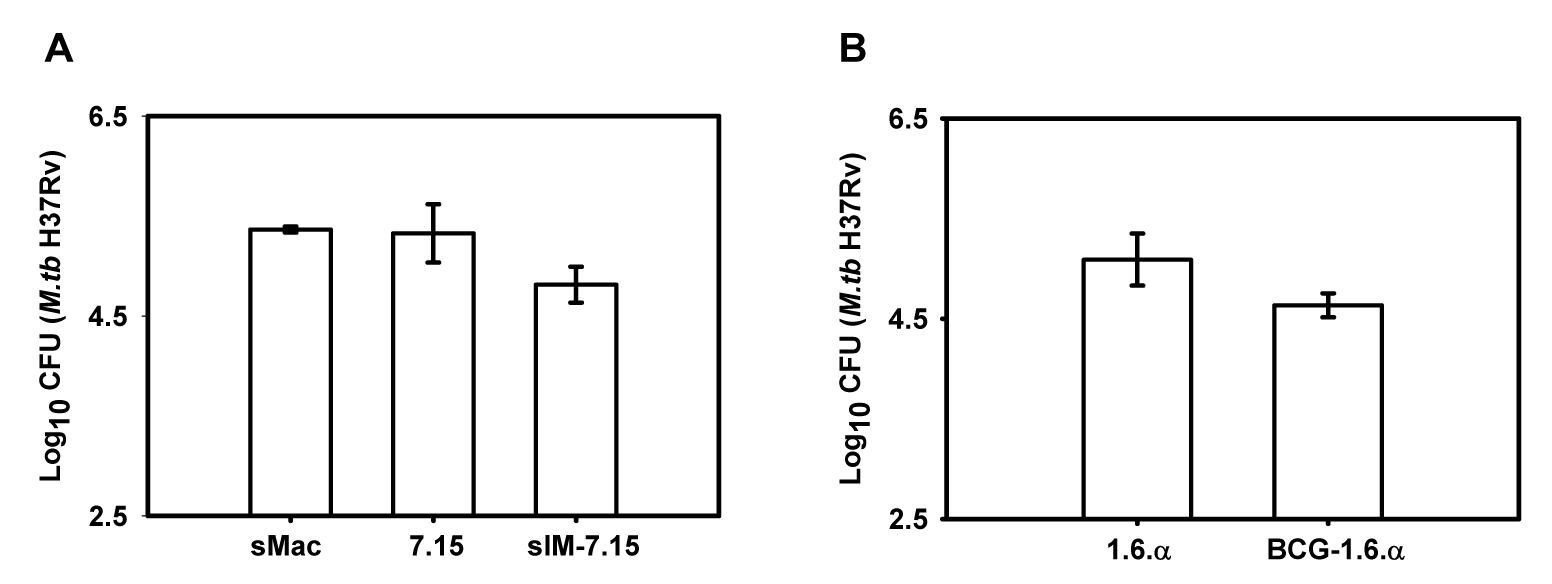

Supplement: Figure S4 — sIM-7.15 and BCG-1.6.α failed to show any protection. sIM-7.15 and BCG-1.6.α vaccinated mice were rested for 240 days before aerosol challenge. After 35 days, animals were sacrificed; mycobacterial load was enumerated by CFU plating. Control groups were immunized with syngeneic uninfected macrophages (sMac), BCG and cytokines alone (1.6.α and 7.15). Data are represented as mean ± SEM of log10 CFU (n = 4–5 animals/group) of two independent experiments. (TIF) [file pone.0016097.s004.tif]

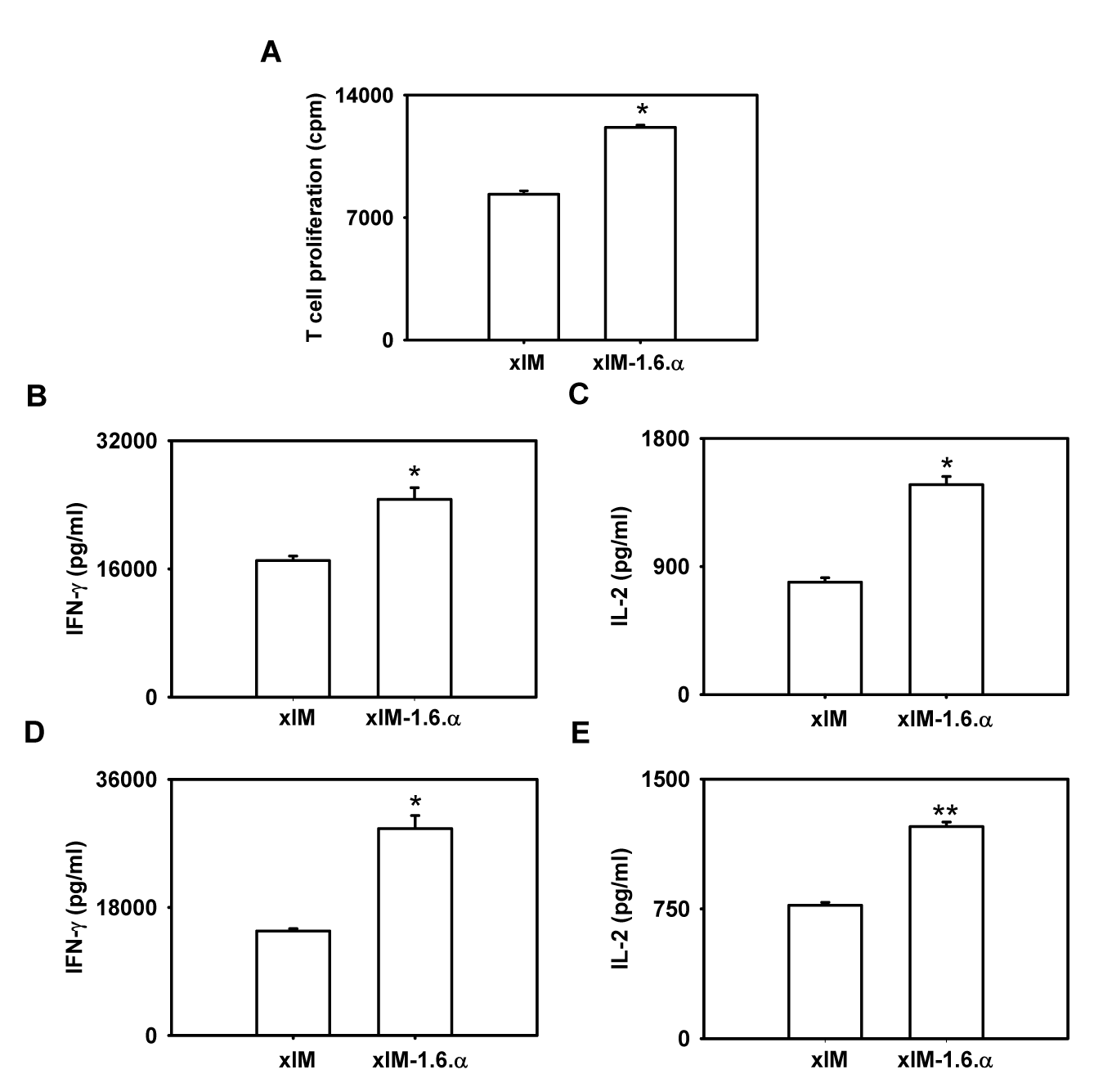

Supplement: Figure S5 — Immunization with xIM-1.6.α also elicits robust immune response against STCF-H37Rv. Mice were vaccinated with xIM-1.6.α and xIM. After 240 days, they were aerosol challenged with M. tb and 35 days later were sacrificed. Lymphocytes Pooled from spleens and lymph nodes (A, B, C) and lungs (D, E) were stimulated in vitro with STCF-H37Rv (50 µg/ml). T cell proliferation was monitored by 3H-thymidine incorporation (A); release of IFN-γ (B, D) and IL-2 (C, E) in the culture SNs by ELISA. Data are shown as mean ± SEM and representative of two experiments, n = 3 animals per group. ‘*’ and ‘**’ indicate p<0.05 and p<0.01 respectively. (TIF) [file pone.0016097.s005.tif]

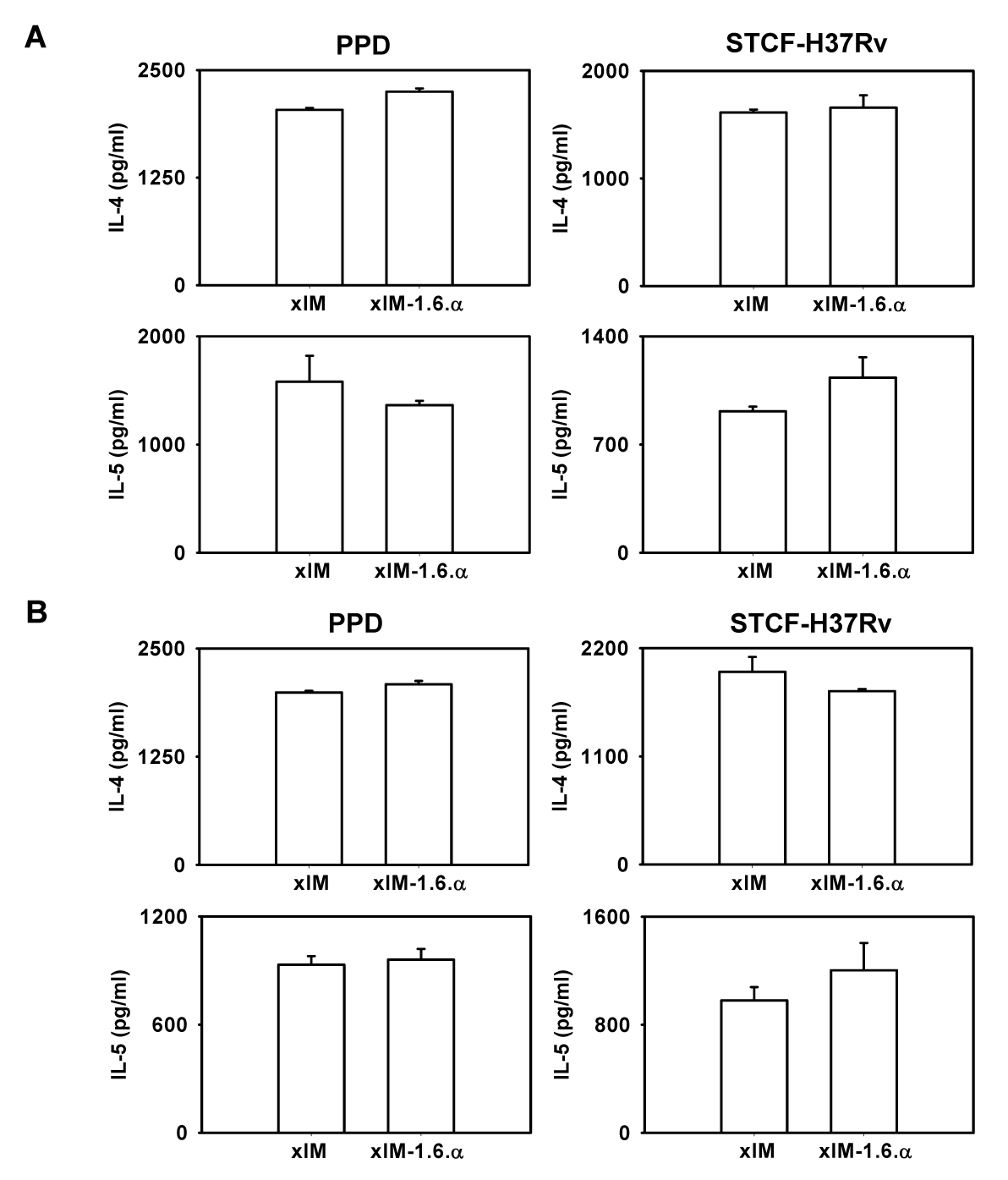

Supplement: Figure S6 — xIM-1.6.α does not augment Th2 response. Mice were vaccinated as mentioned in Figure S5. Lymphocytes pooled from spleens and lymph nodes (A) and lungs (B) were stimulated in vitro with PPD and STCF-H37Rv (50 µg/ml). Secretion of IL-4 and IL-5 was estimated in the culture SNs. Data are shown as mean ± SEM and representative of two experiments, n = 3 animals per group. (TIF) [file pone.0016097.s006.tif]

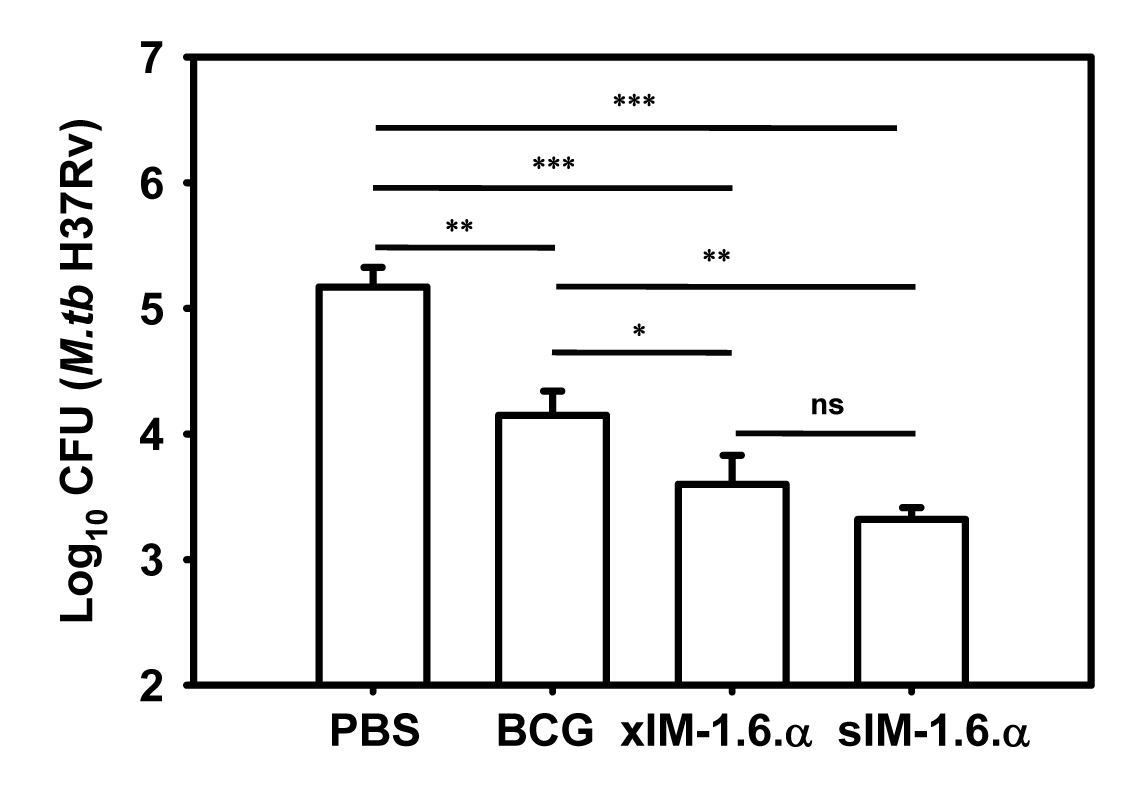

Supplement: Figure S7 — sIM-1.6.α and xIM-1.6.α provides comparable protection against M. tb . Mice were vaccinated with sIM-1.6.α and xIM-1.6.α and rested for 240 days before aerosol challenge with M. tb. After 35 days, mice were sacrificed and mycobacterial load was enumerated by CFU plating of diluted lung homogenates. Control groups were inoculated with PBS and BCG. Data are represented as mean ± SEM of log10 CFU (n = 4–5 animals/group) of two independent experiments. ‘ns’, ‘*’, ‘**’and ‘***’ indicate non-significant, p<0.05, p<0.01 and p<0.001 respectively. (TIF) [file pone.0016097.s007.tif]
